# Systemic endopolyploidy in *Spathoglottis plicata *(Orchidaceae) development

**DOI:** 10.1186/1471-2121-5-33

**Published:** 2004-09-01

**Authors:** Maocheng Yang, Chiang Shiong Loh

**Affiliations:** 1 Department of Biological Sciences, National University of Singapore, 14 Science Drive 4, Singapore 117543

## Abstract

**Background:**

Endopolyploidy is developmentally regulated. Presence of endopolyploidy as a result of endoreduplication has been characterized in insects, mammals and plants. The family Orchidaceae is the largest among the flowering plants. Many of the members of the orchid family are commercially micropropagated. Very little has been done to characterize the ploidy variation in different tissues of the orchid plants during development.

**Results:**

The DNA contents and ploidy level of nuclei extracted from various tissues of a tropical terrestrial orchid *Spathoglottis plicata *were examined by flow cytometry. Sepals, petals and ovary tissues were found to have only a 2C (C, DNA content of the unreplicated haploid chromosome complement) peak. Columns, floral pedicels of newly open flowers and growing flower stems were observed to have an endopolyploid 8C peak in addition to 2C and 4C peaks. In developing floral pedicels, four peaks were observed for 2C, 4C, 8C and 16C. In root tips, there were 2C, 4C and 8C peaks. But in the root tissues at the region with root hairs, only a 2C peak was observed. Nuclei extracted from young leaves shown three peaks for 2C, 4C and 8C. A similar pattern was found in the vegetative tissues of both greenhouse-grown plants and tissue-cultured plantlets. In mature leaves, a different pattern of ploidy level was found at different parts of the leaves. In the leaf tips and middle parts, there were 2C and 4C peaks. Only at the basal part of the leaves, there were three peaks for 2C, 4C and 8C.

**Conclusions:**

Systemic variation of cellular endopolyploidy in different tissues during growth and development of *Spathoglottis plicata *from field-grown plants and *in vitro *cultures was identified. The implication of the findings was discussed.

## Background

In the classical cell cycle, the nuclear DNA contents vary only within the range of 2C and 4C, where C is the haploid DNA content per nucleus. When mitotic DNA replication in somatic cells is not followed by cell division (a process called endoreduplication), variation of cellular ploidy levels (designated as somatic polyploidy or endopolyploidy) can result [1]. Endopolyploidy is considered to be developmentally regulated [2] and has been described in several plant species including maize [3,4], sunflower [5], tomato [6], *Arabidopsis *[7] and brassicas [8,9]. Presence of endopolyploidy as a result of endoreduplication is also a common feature of insects and mammals [10,11].

In orchids, endoreduplication has been described in the raphid crystal idioblasts of *Vanilla *[12] and in parenchyma cells of *Vanda *seedlings [13,14]. The family Orchidaceae has an estimated 17,000 to 35,000 species, making it the largest and an important family of the flowering plants [15]. Many of the members of the orchid family are commercially valuable, and are micropropagated [16]. The explant sources used for orchid micropropagation include inflorescence, leaves, floral buds and roots [16,17]. However, very little is known about the ploidy variation in different explant tissues of the orchid plants during different developmental periods and at the stage when they are used as explants for micropropagation. Increased knowledge of the degree of endopolyploidy in the explant tissue source will be highly valuable for the maintenance of the original ploidy level in culture [8]. In this paper, systemic variation of cellular ploidy and DNA content in different tissues of *Spathoglottis plicata*, a common tropical terrestrial orchid species, from field-grown plants and *in vitro *cultures was investigated.

## Results

### Ploidy level of nuclei isolated from leaves and roots of greenhouse-grown plants

**Figure 1:**
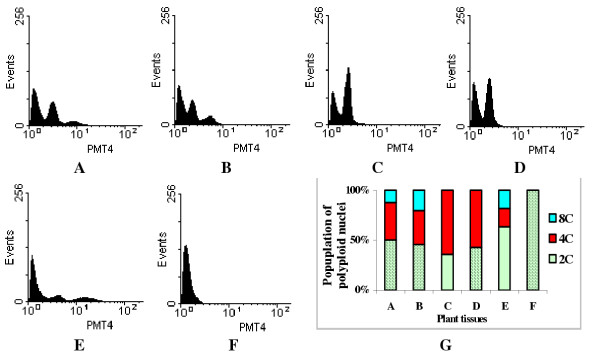
Nuclear DNA content and distribution of endopolyploid nuclei in vegetative tissues of the greenhouse-grown plants: A. Young leaves, B. Basal part of the mature leaves, C. Middle part of the mature leaves, D. Tip of the mature leaves, E. Root tips, F. Root segments with root hairs. The Y-axis presents the number of nuclei (events); the X-axis presents 3-decade log value of relative DNA content (PMT4). G. The population of endopolyploid nuclei in tissues A-F.

Flow cytometry analysis of nuclear preparations from entire young leaves of 1–3 cm in length revealed that there were three peaks of fluorescence corresponding to 2C, 4C and 8C DNA content of somatic cells (Fig. 1A). About 50% of the nuclei were found to have 2C DNA content, 25% were 4C and 15% were found to have 8C DNA content (Fig. 1G).

More detailed analysis was done on mature leaves of 48 cm in length. Tissues taken from different regions of the mature leaves showed that the pattern of ploidy levels was different at different regions. For tissues taken from the basal (petiolar end) of the leaves, there were three fluorescence peaks corresponding to 2C, 4C and 8C nuclear DNA content (Fig. 1B). However, no 8C peak was observed from nuclei preparations taken from tissues of the middle (Fig. 1C) and tip (Fig. 1D) regions of the same leaf; only 2C (35–40% of cell populations) and 4C (60–65% of cell population) nuclei were identified (Figs. 1C,1D,1G).

In the young root tips, 2C, 4C and 8C peaks were observed (Fig. 1E) and the distribution skewed toward 2C population, which accounted for more than 60% of the nuclei population analyzed (Fig. 1G). The percentage of nuclei population with 4C and 8C DNA content in the root tip was relatively small and accounted for only about 10% each (Figs. 1E,1G). Cells from root segments taken at least 2 cm away from the tips were all 2C (Fig. 1F).

### Ploidy level of nuclei isolated from floral tissues

**Figure 2:**
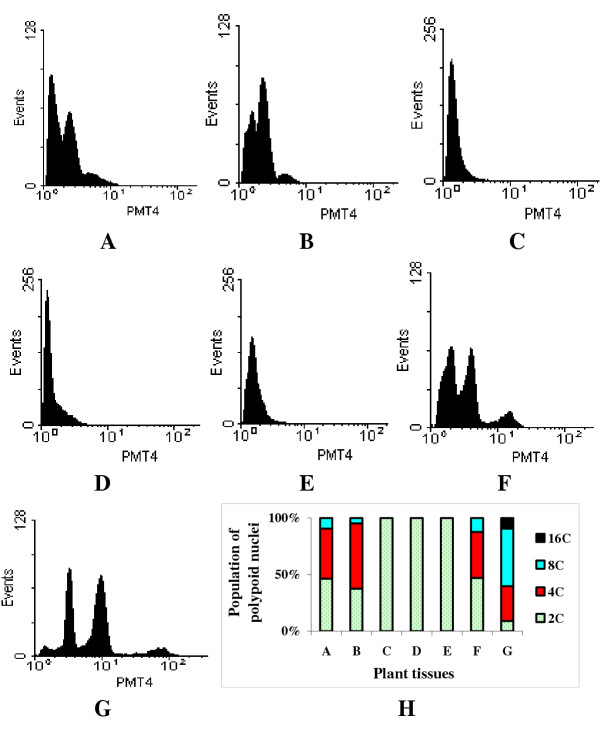
Nuclear DNA content and distribution of endopolyploid nuclei in floral tissues of the greenhouse-grown plants: A. Pedicels, B. Columns, C. Sepals, D. Petals, E. Ovary tissues, F. Growing flower stems, G. pedicels of un-open flowers. The Y-axis presents the number of nuclei (events); the X-axis presents 3-decade log value of relative DNA content (PMT4). H. The population of endopolyploid nuclei in tissues A-G.

Preparations from floral pedicels (Fig. 2A), columns (Fig. 2B) of freshly open flowers, and growing flower stems (Fig. 2F) revealed that there were 2C, 4C and 8C nuclei. The proportions of 2C and 4C nuclei ranged from 40–50% (Fig. 2H), and only about 8 – 10% of nuclei were found to be 8C (Fig. 2H). In pedicels of un-open flower (Fig. 2G), there was a 16C peak in addition to 2C, 4C and 8C peaks. The majority of nuclei were in 4C (28%) and 8C (46%) peaks. The 2C and 16C peaks each had less than 10% of the total nuclei (Fig. 2H). Nuclei isolated from the sepals (Fig. 2C), petals (Fig. 2D) and ovary tissues (Fig. 2E) were all 2C.

### Ploidy level of cells from *in vitro *cultures

**Figure 3:**
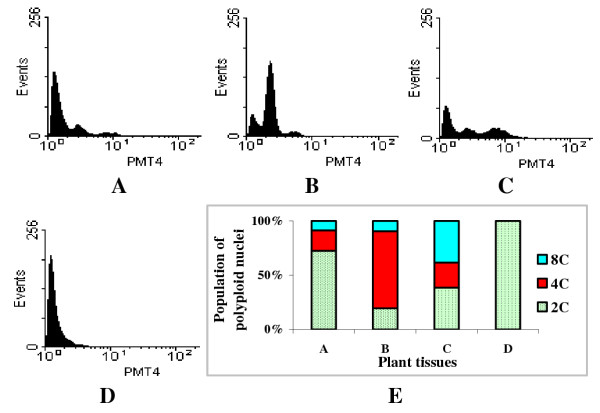
Nuclear DNA content and distribution of endopolyploid nuclei in vegetative tissues of the tissue-cultured plants: A. Protocorms, B. Young leaves, C. Root tips, D. Root segments with root hairs. The Y-axis presents the number of nuclei (events); the X-axis presents 3-decade log value of relative DNA content (PMT4). E. The population of endopolyploid nuclei in tissues A-D.

Protocorms of *S. plicata *were found to have 2C, 4C and 8C nuclei (Fig. 3A) with majority (over 70%) of them with 2C DNA content (Fig. 3E). In the young leaves of plantlets, majority (70%) of the nuclei isolated were 4C, and about 20% of were 2C nuclei and the rest 8C (Figs. 3B,3E). In the root tips of cultures, there were about 40% each of 2C and 8C nuclei, and the proportion of 4C nuclei was only about 10% (Figs. 3C,3E). Nuclei taken from root tissues at the region with root hairs were all 2C (Figs. 3D,3E).

## Discussion

As references to the DNA content of gametic nucleus of individuals, DNA 'C' values have been estimated in several thousand animal and plant species [18]. For angiosperms, information on 'C' values is used in a wide range of biological fields [19]. The 1C DNA values in angiosperm plants differ approximately 1000 folds, ranging from 0.13 pg in *Arabidopsis thaliana *to 127.4 pg in *Fritillaria assyriaca *[18]. The DNA content per genome is usually considered to be constant between cells in an individual, and relatively constant between individuals of the same species [18]. However, in some plant species, intraplant ploidy variations were reported, and this implied that the nuclear DNA content in these species is not static and hence a great amount of variation occurs [8]. For example, a survey of *Arabidopsis thaliana *revealed endopolyploidy in hair trichomes, leaf epidermal cells, root tip cells, and cells in the hypocotyls [7,20], but not in the inflorescence [7]. In some cell types, the extent of endoreduplication appears to be intrinsically controlled by the differentiation programme, but environmental influences such as light can also affect endoreduplication [21].

The patterns of endopolyploidy may be affected by plant growth conditions in some plants. For example, leaves of *in vitro *grown tomato and potato plants were found to have lower level of endopolyploidy than leaves of plants grown in the greenhouse [22,23]. However, in *S. plicata*, patterns of endopolyploidy were found to be similar in both tissue-cultured plants and greenhouse-grown plants. Endoreduplication was found to occur in actively growing tissues with of *S. plicata *such as young leaves (1–3 cm in length, newly initiated) and root tips from greenhouse-grown plants. Similarly, endopolyploid cells were found in protocorms, young leaves and root tips from *S. plicata *seedlings in tissue culture. The common feature for protocorms, young leaves of 1–3 cm and root tips are that they are young and active in cell division and growth. In other orchids such as *Dendrobium*, endopolyploidy was found in root tips and newly expanded young leaves [24]. In the root segment with root hairs of *S. plicata*, endopolyploidy was neither found in tissue-cultured plants nor it was detected in greenhouse-grown plants. These results imply that the presence of the endopolyploidy during *S. plicata *development is an intrinsic programme, and it is not much affected by the growth condition.

Besides root tips and newly developing young leaves, endopolyploidy was observed in mature leaves in a few *Dendrobium *species and cultivars [24]. Endopolyploidy was also detected in mature leaves of *S. plicata. *Furthermore, when the tip, middle and basal parts of the mature leaf were examined, different patterns of ploidy levels were obtained. Endopolyploidy was found only in leaf base part of mature leaves. A mature leaf represents a continuous developmental system, with the young, less green meristem cells at the basal petiolar end and the older, photosynthetically active cells at the tip [25]. Previous research shown that in cucumber and succulent plants with small genome, the level of endoreduplication does not increase once an organ is fully developed [26,27]. The tips and middle parts of mature leaves in *S. plicata *are fully developed. Endoreduplication in these tissues is unlikely since it would lead to further cell expansion. The pattern of DNA ploidy variation within the mature leaf is closely associated with the developmental status. The mechanism that resulted in endopolyploidy, however, remains unclear.

In *S. plicata*, endopolyploidy was present in some floral tissues such as columns, growing flower stems and pedicels of both un-open and freshly open flowers. However, other floral tissues like sepals, petals and ovary tissues were found to have only 2C nuclei. In the growing un-open flower pedicels, the highest ploidy level even reached 16C. In cabbage, endopolyploidy was reported in cabbage flowers [8], and detailed patterns of endopolyploidy were found in various developmental stages of petals [9]. In cabbage petals, differentiation of expanding cells was characterized by endoreduplication [9]. In the proximal part of the cabbage petal, differentiation was accompanied with endoreduplication and cell enlargement. By contrast, no endopolyploid nucleus was found in the distal part of the lamina in the mature cabbage petal [9]. This study suggested that the developmental program of the cabbage petals might induce the initiation of endoreduplication [9]. In *Arabidopsis*, endopolyploidy was found in hypocotyls, cotyledonary leaves, rosette leaves, stems of bolting plants and floral leaves, but was not found in inflorescences [7]. Given the small size of columns within *Arabidopsis *floral buds, and the small population of endopolyploid nuclei found in columns and pedicels of *S. plicata *in this study, the minute population of the endopolyploid nuclei could easily be neglected when the whole floral buds were used for sampling. In *S. plicata*, it was found that the average size of nuclei was larger in columns and pedicels that have a measurable amount of endopolyploid cells than in other flora tissues without endopolyploidy (unpublished results). Similarly, a correlation was found between cell size and ploidy levels during cabbage petal development [9]. In *Dendrobium*, the post-pollination physiological changes were found to be different between floral tissues such as columns, ovary tissues, sepals and petals [28]. Edgar and Orr-Weaver [10] suggested that as endoreduplication is often found in large cells or cells with high metabolic activity, it might be a common strategy for cell growth without division.

Further evidence was found in legumes where cell differentiation to a specialized function as pod wall tissues was accompanied by endoreduplication, and higher ploidy levels coincided with maximum pod growth [29]. During tomato fruit development, the pericarp tissue of young green fruit did not have higher ploidy (usually within 2C and 4C), but most of the cells in pericarp became endopolyploid (up to 256C) as the fruit developed further [6]. In tobacco single cell culture, endoreduplication was associated with plant growth regulators. When auxin was applied alone, endoreduplication was induced and the DNA content kept pace with the increment of cell volume. When both auxin and cytokinin were supplied subsequently, the cells divided first as amitosis leading to DNA endoreduplication, then followed by normal mitosis cell cycles [30]. Gibberellin and ethylene were found to play important roles in the endoreduplication of *Arabidopsis *hypocotyls [31]. In cabbage, mammals, *Drosophila melanogaster *and some small genome plants like *Arabidopsis*, it is thought that endoreduplication is developmentally regulated [8,10,32].

The systemic endopolyploidy revealed within different tissues of *S. plicata *raises the question of its possible implications. In tobacco, it was reported that the morphogenetic response of the tissues culture was related to the nuclear DNA content variation within stem explants of different ages [25]. In *Oncidium *Gower Ramsey, a hybrid orchid, only root tips, cut surfaces of stem segments and young leaves were able to form callus in tissue culture. Other explants such as old leaves and the roots without meristem tips could not form any callus [33]. Molecular data showed that the nuclear DNA modulation was closely related to the acquisition of embryogenic competence in cultured carrot hypocotyls [34]. In various tissues in cabbage plants, the number of endocycles was tissue-specific and was characteristic of the developmental stage [8,9,32]. These studies suggested that pattern of endopolyploidy may represent the characteristic of the developmental and physiological properties of the tissue.

The role of endoreduplication in plant development is still not well understood. The presence of endopolyploidy was proposed to be associated with several factors, such as taxonomic position of a species, life cycle, genome size, and organ type [35]. Recent investigation of 16 plant species suggested that endopolyploidization might provide a mechanism to facilitate plant growth [35]. Endoreduplication benefits fast growth in several ways. In polyploid cells, the increased gene dosages may enhance the transcriptional and metabolic activities. In addition, several processes are eliminated in the endoreduplication cycle such as the reorganization of the cytoskeleton and condensation of the chromosomes, and that might allow faster growth [36]. Ploidy level also plays a role in controlling the size of the cells, the organs or the whole plant [6,9,37]. One of the common features of plant development is the uneven enlargement of plant cells coupled to somatic endoreduplication, which indicates that the enlargement of plant cells might be the consequence of the increased genome size [9,37].

This research may also have an impact on the orchid industry. Orchidaceae is the largest family of the flowering plants, and many of its members are commercially hybridized [38]. Clonal propagation is a common and essential practice for multiplication of hybrid orchids because the genotypes of the hybrids are usually heterozygous [39]. Many tissues have been used as explants for micropropagation including inflorescence, leaves, floral buds and roots [16,40,41]. Somaclonal variation is undesirable, and it is a major problem encountered in commercial micropropagation of orchids if true-to-type plants are required [39,42]. The mechanism of the somaclonal variation is poorly understood [42]. Polyploidy is considered as a possible cause for somaclonal variation in tissue cultures [43], but how polyploidy is generated during tissue culture is unclear [39,42]. The presence of systemic endopolyploidy and DNA content variation within different tissues of *S. plicata *as revealed in this study suggests that endopolyploidy and DNA content variation in explants might be a cause for somaclonal variation in tissue culture derived orchid plantlets. Thus, the pre-knowledge about the ploidy variation in different explant tissues is valuable for clonal propagation or for deliberate induction of variants in culture. Further systemic investigation of the relationship between somaclonal variation and type and endopolyploid level of source explants will provide indepth knowledge for micropropagation of orchids.

## Conclusions

Systemic variation of cellular endopolyploidy in different tissues during growth and development of *Spathoglottis plicata *from field-grown plants and *in vitro *cultures was developmentally regulated. Pattern of endopolyploidy is a character of the developmental and physiological properties of the tissue. This finding provides useful information for understanding of the plant development and for industrial propagation of orchids.

## Methods

### Plant materials

*Spathoglottis plicata *L. is a common tropical terrestrial orchid. The plants were grown in pots and placed in the greenhouse at 28 ± 4°C without artificial lighting. The following materials were taken for analysis: a) young leaves (1–3 cm in length), b) mature leaf (48 cm in length), c) root tips (2 cm including the tip), d) root segments from region with root hairs (2 cm away from the root tip), e) newly opened flowers, f) growing flower stems and g) developing floral pedicels of un-open flower (4–6 days before flowering).

Seedpods were surface-sterilized for 20 min in 20% (v/v) Clorox™ solution and subsequently rinsed 3 times with autoclaved water. Seeds were germinated aseptically in 9 cm diameter petri dishes containing 25 ml Knudson C orchid medium (Duchefa, Netherlands) with 2% (w/v) sucrose and 0.8% (w/v) agar. All cultures were incubated at 25 ± 2°C under a 16 h photoperiod (light intensity: 54 μm^-1^m^-2 ^s^-1^). The following materials from *in vitro *cultures were examined: a) protocorms (6 weeks after germination), b) young leaves (1–2 cm in length) from seedlings, c) root tips (1 cm segments from tips), d) root segments with root hairs (at least 1 cm away from the root tips).

### Preparation of nuclei and flow cytometry analysis of nuclear DNA content

Extraction of nuclei and staining of DNA were performed according to the method of Arumuganathan and Earle [44,45] with some modifications. All preparations were done on ice. Tissues (about 0.3 – 1.0 g) were sliced with razor blades into strips of less than 1 mm in 1 ml extraction solution (1 mM MgSO_4_, 5 mM KCl, 0.5 mM HEPES, 1 mg/ml dithiothreitol, 2.5 mg/ml Triton X-100, pH 8.0) and extracted for 45 minutes. After filtering through a 45 μm Falcon cell strainer, 100 μl of propidium iodide (1 mg/ml) and 2.5 μl of 500 μg/ml DNase-free RNase (Boehringer Mannheim, Indianapolis, IN) were added to each sample followed by 30 min incubation at 37°C.

A Coulter EPICS^® ^Elite ESP with 15 mW 488 nm Cyonics Argon air-cooled laser flow cytometer was used to measure the relative fluorescence of nuclei. For each sample, at least 10,000 nuclei were analyzed. Data were analyzed with WinMDI27B software (Joseph Trotter™).

## Abbreviations

C, DNA content of the unreplicated haploid chromosome complement

PI, propidium iodide

PMT4, photo multiplier tube

## Authors' contributions

MY carried out experiments in the project. CSL and MY prepared the manuscript.
